# Toxic Metals and Metalloids in Hassawi Brown Rice: Fate during Cooking and Associated Health Risks

**DOI:** 10.3390/ijerph191912125

**Published:** 2022-09-25

**Authors:** Abdulaziz Abdulrahman AlMulla, Saad Dahlawi, Muhammad Atif Randhawa, Qamar uz Zaman, Yinglong Chen, Turki Kh. Faraj

**Affiliations:** 1 Department of Environmental Health, College of Public Health, Imam Abdulrahman Bin Faisal University (IAU), P.O. Box 1982, Dammam 31441, Saudi Arabia; 2 Department of Environmental Sciences, The University of Lahore, Punjab 54590, Pakistan; 3 The UWA Institute of Agriculture, and School of Agriculture and Environment, The University of Western Australia, Perth, WA 6009, Australia; 4 Department of Soil Science, College of Food and Agriculture Sciences, King Saud University, P.O. Box 145111, Riyadh 11362, Saudi Arabia

**Keywords:** brown rice, heavy metals, environmental pollutants, daily intake, health risk

## Abstract

Rice has been a dietary staple for centuries, providing vital nutrients to the human body. Brown rice is well known for its nutrient-dense food profile. However, owing to multiple causes (anthropogenic and non-anthropogenic), it can also be a potential source of toxic heavy metals in the diet. Brown Hassawi rice samples were collected from the Al-Ahsa region and analyzed for its content of toxic metals. The results reveal that all the tested metals varied significantly in the brown rice samples, while As and Pb in all three samples exceeded their respective maximum allowable limits (MALs), followed by Cd, which nearly approached the MAL in two samples out of three. Brown rice samples were cooked in rice:water systems, viz., low rice:water ratios (1:2.5, 1:3.5) and high rice:water ratios (1:5, 1:6), along with soaking as a pre-treatment. Soaking was unproductive in removing the heavy metals from the rice, whereas cooking dissipated all metals from the rice, except for Cd, which was statistically non-significant. The high-water cooking of the rice was more effective in the dissipation of metals from the rice as compared to low-water cooking conditions. Through the consumption of rice, the estimated daily intake (EDI) of heavy metals is 162 g per person per day for As, which is above the provisional maximum tolerable daily intake (PMTDI) regardless of cooking circumstances. The hazard risk index (HRI) also highlighted the fact that As can be a potential health hazard to rice consumers in the Al-Ahsa region of Saudi Arabia. These results indicate the potential health risks caused by the consumption of this rice by humans. Regular monitoring is recommended to manage and control elevated concentrations and related health hazards as a result of the use of Hassawi rice contaminated by the accumulation of metals and metalloids.

## 1. Introduction

For centuries, cereals such as rice, wheat, corn, barley and sorghum have been considered dietary staples for humankind. Among these, rice (*Oryza sativa* L.) is the most important cereal crop which is consumed by over half of the world’s population as a staple food to meet daily dietary requirements [1]. The major rice-producing countries are in the Asian region of the world and include China, the Indo-Pak subcontinent, Indonesia and Korea, and diverse types of rice varieties are cultivated in these countries [2]. World rice production in 2019 was reported to be 782 million tons on a 167.13 million-hectare cropping area [3]. However, evidence shows that rice is also cultivated in some parts of western Asian countries such as Iran, Iraq, Egypt and even the Kingdom of Saudi Arabia, with a total production of 1.226 million tons against a 0.179 million-hectare area in 2019 [3]. In Saudi Arabia, the Al-Ahsa region is well known for rice cultivation, and the indigenous rice or landrace adapted in this part of the world is known as Al-Ahsawi or Hassawi rice [4]. Hassawi rice is resistant to salinity and drought, thereby enabling it to tolerate adverse environmental conditions, and it also has the ability to survive under harsh agro-environmental conditions [5]. However, low resistance to lodging, photoperiod sensitivity and water scarcity in the region are hindering the widespread cultivation of Hassawi rice, although it has been cultivated for centuries in this part of the world [6,7].

Hassawi rice is eaten in its whole form with intact outer bran layers that contains many nutritional compounds [8,9] in the Kingdom of Saudi Arabia. At present, food scientists/nutritionists are encouraging the consumption of brown rice over the processed white type of rice due to its nutritional significance [8]. Brown rice contains more proteins and a sufficient quantity of micronutrients, making it a perfect food for human beings [8]. Hassawi rice is traditionally well known by the masses for its property of providing strength to those who are unwell [4]. In addition to its high nutritional properties, brown rice can be a potential source of toxic metals [10]. Toxic metals in rice are becoming a global problem day by day due to increased urbanization, intensive agricultural operations, sewage sludge applications to soil, mining activities, etc. [11].

Rice is more likely to accumulate heavy metals as compared to other cereal grains, particularly cadmium and arsenic [12]. Under rice-growing conditions, arsenic is readily converted into arsenite, thereby enhancing the accumulation of arsenic in rice grains by up to 10-fold as compared to other staple crops [13]. Lead is the second most common element with respect to natural occurrence after arsenic, and both are highly toxic to human beings, particularly when exceeding their maximum allowable limits (MALs) in foods [14]. These may also stress rice plants as they are growing, which could result in an expansion of their tissues and ultimately have a detrimental impact on consumers [15,16,17]. Brown rice can accumulate a higher level of toxic metals such as arsenic, cadmium and lead, and long-term exposure to heavy metals through brown rice consumption poses both non-carcinogenic and carcinogenic health risks to consumers [18,19,20]. Humans are usually exposed to heavy metals through dietary intake, particularly when metal concentrations in foods exceed their MALs, as well as through ingestion or breathing. Because heavy metals are known to be mutagenic, teratogenic and carcinogenic, consuming them causes the onset of many disorders [21]. In connection to human exposure, the hazards of ingesting heavy metals include cirrhosis, dementia, encephalopathy, irreversible brain damage, bleeding, renal dysfunction, bone disorders, sperm motility, cardiovascular illnesses and gastrointestinal malignancies [22,23].

Very limited studies are available in the literature on the fate of toxic heavy metals during food cooking operations. Perello [24] studied the effect of food processing unit operations on concentrations of arsenic, mercury, cadmium and lead in food products. They found that cooking operations exerted a limited impact on the removal of heavy metals from the foods under investigation and concluded that the hypothetical reduction in heavy metals depends upon the cooking conditions (time, temperature and medium of cooking) [25]. Some studies on the impact of rice cooking conditions on arsenic levels have been carried out by a few researchers [26,27]; however, the impact of cooking operations on other toxic metals is still lacking in the literature, particularly for brown rice. Furthermore, to the best of our knowledge, there is no research on the presence of heavy metals and metalloids in brown Hassawi rice produced in the Saudi Arabian Al-Ahsa region. Therefore, the objectives of this study were to (1) ascertain hazardous metals and metalloids in brown Hassawi rice; (2) evaluate how metals and metalloids behave under various rice cooking conditions; and (3) document, for the first time, the health hazards associated with brown Hassawi rice consumption.

## 2. Materials and Methods

### 2.1. Study Area

Al-Ahsa has a dry, tropical climate, with a five-month summer and a relatively cold winter. It enjoys the benefit of copious reserves of underground water, which have allowed the area to develop its agricultural potential. The land of the Kingdom of Saudi Arabia is mostly arid, and some parts of the eastern side have fertile land that is used for crop and vegetable production. Based upon these observations, two farms were selected for the rice kernel sampling.

### 2.2. Hassawi Rice Procurement

Traditionally grown rice from the Al-Ahsa region of the Kingdom of Saudi Arabia (Hassawi rice) was procured during 2019 from 2 rice farms, and one sample was procured from a fruit and vegetable market in Al-Hofuf. From each farm, 05 various points were selected, and from each point, 03 samples were collected from three different locations. On the basis of the availability of these rice samples, composite samples were prepared. Each composite sample (4 kg) was procured in a sealed envelope and brought to the laboratory (Environmental Health Laboratory, College of Public Health at Imam Abdulrahman Bin Faisal University, Dammam, Saudi Arabia) for analysis. After sun drying, the kernel samples were moved to the oven and dried at 105 °C for five days.

### 2.3. Toxic Metals Determination

Exactly 0.2 kg of rice from each sample was used for heavy metal determination, and the remaining samples were stored under shade in the laboratory for later studies. The oven-dried brown rice samples were ground with the help of a laboratory grinder equipped with a stainless-steel blade. Ground rice powder (2 g) was digested using a mixture of di-acids (HCl and HNO_3_, Sigma-Aldrich, St. Louis, MO, USA) in 12 mL at 2:l *v*/*v*. In a microwave-accelerated reaction system (MARS 6), the mixture was heated for 45 min at a maximum temperature of 180 °C and a maximum pressure of 200 psi [28]. After the solution was digested, Whatman No. 42 ash-free filter paper was used to pass the solution by adding 25 mL of deionized water. The samples were kept until analysis in polyethylene bottles that were already cleaned and acid-washed. Metal and metalloid analysis was conducted on an iCAP 6300 Duo Inductively Coupled Plasma–Optical Emission Spectrophotometer (ICP-OES, Thermo Fischer Scientific, Waltham, MA, USA) following the protocols of [29]. To obtain precise outcomes, the chemicals and salts required for digestion were obtained from E. Merck (Darmstadt, Germany) with a certified purity of 99%. Standard preparation precautions were sustained during the experiment. Metal absorption in rice was determined on a dry weight basis. Each sample was analyzed three times. The findings of the experiments on the presence of heavy metals and metalloids in rice are presented as the mean and standard deviation. Based on the findings of the heavy metal analysis of the rice samples, one rice sample was selected for determining the fate of metals during cooking, and for the assessment of health risks to consumers due to rice consumption.

### 2.4. Fate of Toxic Metals during Rice Cooking

The selected rice sample was used for the estimation of the fate of heavy metals in brown Hassawi rice during cooking under different rice:water systems in triplicate, as detailed below. Brown Hassawi rice was cooked under laboratory conditions with different rice:water ratios during cooking and compared with raw rice as a control (T_0_), T_1_ = soaking of rice in ample water for 20 min and different cooking treatments, viz., T_2_ = (1:2.5), T_3_ = (1:3.5), T_4_ = (1:5) and T_5_ = (1:6) rice:water ratios. An amount of 100 g of rice was used in each cooking experiment, and the rice was soaked for 20 min in an ample quantity of double-distilled water before cooking as per routine practice followed in the home cooking of brown rice. Afterwards, the rice was cooked in a stainless-steel pan (14 cm diameter) for 40 min. In the low-water system (T_3_ and T_4_), all water was absorbed by the rice during cooking, whereas the extra water in the high-water cooking system (T_5_ and T_6_) was collected as well as analyzed for the possible presence of heavy metals. Both the cooked rice and drained water were collected and preserved in high-density polyethylene bags and kept under refrigerated conditions until further processing. The rice samples were dried in an oven until constant weight and then analyzed for metals as stated above.

### 2.5. Dietary Intake Assessment

In the Kingdom of Saudi Arabia, rice is a staple food. To determine the daily intake of metals and metalloids from rice consumption, the concentration of a particular metal was multiplied by the per capita daily consumption of rice and divided by the average adult human body weight [30]. Calculated daily intakes of toxic heavy metals and metalloids from eating rice were compared to the suggested values of [31]. According to [3], per capita rice consumption in the KSA is 162 g/person/day, and the average body weight of a Saudi citizen is 71 Kg [32]. Thus, daily consumption values for rice cooked in low-water and high-water boiling systems were computed. The information was compared to the provisional maximum tolerated daily intake (PMTDI).

### 2.6. Health Risk Index

The health risk index for the consumption of rice was calculated by following the guidelines of [30]. The health risk index of heavy metals and metalloids in the rice was calculated using the daily intake assessment values of heavy metals and metalloids. HRI values can be calculated by dividing the daily intake of metals and metalloids in rice by the oral reference dose:

HRI = DIM/RfD

HRI > 1 for any metal means that the consumer’s/population’s health is at risk.

### 2.7. Statistical Analysis

Data collected in the experiment were processed by employing the descriptive analysis technique. All data processing and statistical calculations were carried out using statistical software (Statistix, Version 10), and the analysis of variance technique for comparison of treatment means was carried out using XLSTAT software. All the presented figures were created using Sigmaplot 12.5 (SPSS Inc., Chicago, IL, USA).

## 3. Results

### 3.1. Screening of Toxic Elements from Rice

For screening of toxic elements, raw Hassawi rice samples were analyzed for possible contamination with toxic metals. The statistical results presented in Table 1 clearly show that the occurrence of heavy metals varied in the rice samples with respect to the variation in the field. All the samples exceeded the maximum allowable limits (MALs) prescribed by the FAO/WHO for arsenic and lead contents [31]. The Cd and Cr contents were within their MALs prescribed by the FAO/WHO for rice/food stuffs intended for human consumption [31]. However, the FAO/WHO [31] have not defined/established MALs for Sb and Ni in rice/food stuffs. The highest (mg/kg) arsenic (0.476), Cd (0.374), Cr (0.386) and Ni (1.386) contents were found in the rice sample procured from farm 2. Keeping these facts in view, the rice sample from farm 2 was selected for further studies and the estimation of health risk assessments, as detailed in the methodology section of this study.

### 3.2. Effect of Cooking on Heavy Metals in Rice

The mean square of the effect of cooking conditions on heavy metals in rice is shown in Table 2. It is clear from the statistical results that cooking conditions (such as the rice:water ratio system) significantly affected all the tested metals’ (As, Pb, Cr, Sb and Ni) contents in brown Hassawi rice, except for Cd, which was non-significant.

**Figure 1:**
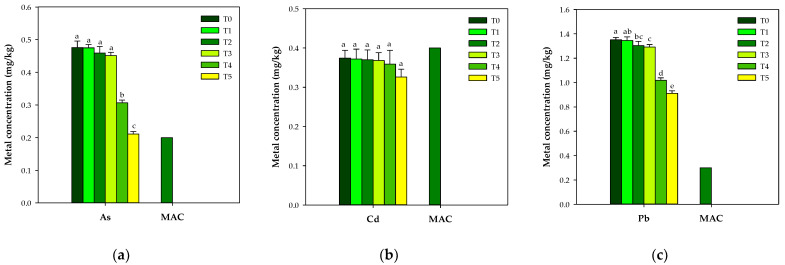

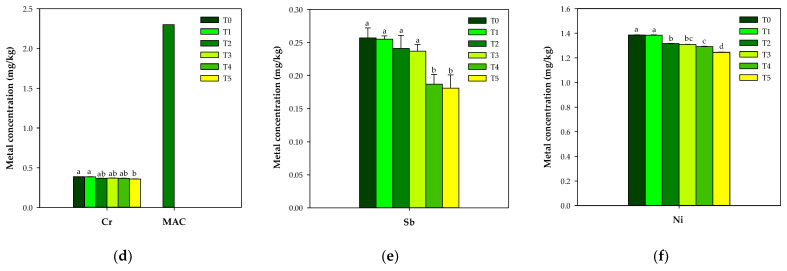
Effect of cooking treatments on As (**a**), Cd (**b**), Pb (**c**), Cr (**d**), Sb (**e**), and Ni (**f**) contents in Hassawi rice; similar letters on bars are non-significant among each other (*p* > 0.05); MAC means maximum allowable concentration by FAO/WHO; for Sb and Ni, the maximum allowable concentration is not defined.

The soaking of rice for 20 min in sample deionized water did not affect the toxic metal contents, and its impact on the reduction in heavy metals in the rice was almost zero. Meanwhile, different behaviors were observed for cooking under a low-rice:water system (T_2_ and T_3_) and cooking under a high-rice:water system (T_4_ and T_5_). In general, the rice cooking treatments T_4_ and T_5_ resulted in the greater removal of heavy metals from the rice as compared to T_2_ and T_3_ (Figure 1).

**Figure 2:**
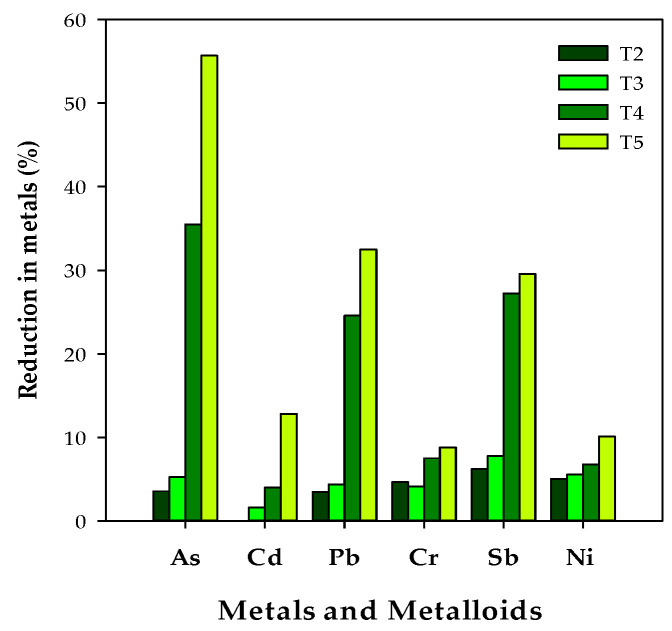
Reduction in metals during rice cooking in low- and high-water system.

The arsenic contents in the cooked rice (T_4_ and T_5_) were more significantly affected among all the metals in this investigation. Arsenic was reduced by up to 55% in the cooked rice as compared to the arsenic contents in the raw rice. Lead contents were decreased by 32.44% (Figure 2) in the T_5_ treatment, and this reduction in arsenic contents can be attributed to the dissolution of arsenic in the excess water used under cooking conditions, which ultimately resulted in the removal of arsenic from the rice. However, in T_2_, no excess water was available, and in T_3_, the excess water was evaporated to dryness whilst the rice was cooking. Although Cd contents decreased more in the high-rice:water system (T_4_ and T_5_), the decrease was non-significant as compared to As and Pb. Cadmium was absorbed by the roots and deposited in the endosperm, which ultimately makes its removal difficult during rice cooking.

### 3.3. Estimated Daily Intakes of Metals from Hassawi Brown Rice

The estimated daily intakes (EDIs) of several toxic heavy metals and metalloids from eating rice are shown in Table 3. In 2015, the FAO Stats reported that Saudi Arabians consumed 162 g of rice per person per day. For each metal and metalloid found in the rice, the predicted daily intakes of heavy metals and metalloids were calculated. Table 3 also displays the permitted maximum tolerated daily intake (PMTDI) values for the various toxic metals under consideration. The results show that all the heavy metals except for arsenic were within the safe limit of consumption with respect to their PMTDIs. The EDI of arsenic was estimated to be 74.35 and 73.06 (µg/day) in T_2_ and T_3_, respectively. However, cooking the rice with excess water in the high-water system and draining the excess water from the rice resulted in a significant reduction in arsenic intake through the rice. The arsenic contents decreased to 49.73 and 34.18 (µg/day) in T_4_ and T_5_, respectively. The arsenic contents decreased by approximately 55%; however, the arsenic’s EDI was still higher than the PMTDI (25.2 µg/day). This means that the cooking rice in a high-water system is desirable to remove toxic elements from the rice. This is also a common practice in some households, where rice is boiled in excess water, the unnecessary water is removed, and the rice is cooked to the desired degree.

### 3.4. Health Risk Index of Metals from Rice Intake

As indicated in Table 4, the health risk index (HRI), which was calculated based on the EDIs and oral reference doses of each toxic metal, was used to figure out the actual health hazards. With regard to cadmium, lead, chromium, antimony, and nickel, the results show that there is no health risk associated with consuming brown Hassawi rice because their calculated values for the HRI were less than 1.00 (permissible limit). The high-water cooking of the rice and the drainage of excess water from the rice during cooking provided better results in reducing the health risks. However, arsenic in brown rice may pose potential health hazards to brown rice consumers. Although arsenic contents were significantly reduced during rice cooking in the different treatments, its health risk value was still more than 1. In the case of arsenic, the T_5_ cooking treatment was most effective, and it decreased the health risk value from 3.62 (raw) to 1.61, meaning that it reduced the health risk. However, the health risk increases when the metal intake comes from multiple sources in the form of combined exposure to metals.

## 4. Discussion

Industrialization and the agriculture sector are considered the backbone of the economy of any area [39]. However, these sectors significantly contribute to the soil pollution in that area. Moreover, anthropogenic activities are also the main reason for soil and groundwater pollution [40]. The production of food on these polluted soils or using metal-contaminated irrigation water leads to the bioaccumulation of metals and metalloids in food, which creates severe health complications in the local community feeding on that polluted produce [41].

Because of insufficient waste disposal in the environment, land, air, and water are becoming more and more contaminated as a result of the nation’s fast industrialization [11,42]. Crops are also becoming more and more polluted as a result of soil contamination. Industry is the first link in the transmission chain, followed by the environment (soil and water), foods (crops and vegetables) and, finally, people [43]. When food is consumed, heavy metals such as Pb, Cd, Mn, and As can enter the body through the mouth and gastrointestinal tract, causing major health problems. Heavy metals can also enter the body through breathing [44].

The results of the present study are comparable with those of previous studies, which have reported that the levels of arsenic, lead, and cadmium ranged from 0.21 to 0.98, 0.04 to 1.07, and 0.006 to 0.24 mg/kg, respectively, in brown rice near a mining area in Central China [19]. In a recent investigation of some imported rice varieties sold in the local markets of AlMadinah AlMunawarah, KSA, the results revealed that the country of origin affected the concentration of toxic metals (arsenic, cadmium, lead, and chromium) in rice grains, and the authors urged the regulatory authorities to monitor the heavy metal contents of rice imported in the Kingdom of Saudi Arabia [45,46]. The results clearly elucidated that both arsenic and lead contamination in brown rice can be a threat to human beings [44]. Although cadmium contamination in rice is within safe limits, it is at the marginal level and nearly approaching the upper safe limit. Arsenic contamination in rice is a natural process, particularly if arsenic-contaminated water is used for irrigation or the soil itself has a high load of arsenic due to the nature of rock formation [47]. It has already been proven that, under rice-growing conditions, arsenic is readily converted into arsenite, thereby enhancing the accumulation of arsenic in rice grains by up to 10-fold as compared to other staple crops [13,48]. Furthermore, anthropogenic activities also lead to the deposition of arsenic in the soil, such as smelting and mining processes, and the use of arsenic as an active ingredient in insect pest control programs in agriculture for crop protection [49]. Fu et al. [50] reported heavy metal contamination in brown rice from three districts of Hunan Province in China and found that the arsenic level ranged from 0.106 to 1.15 mg/kg, with a mean value of 0.336 mg/kg. In China, the MAC of arsenic in brown rice is 10 mg/kg [33]. When comparing with the Chinese MAL value set for arsenic contents in rice, Hassawi rice looks to be safe for human consumption, despite exceeding the FAO/WHO maximum allowable limit [31].

Lead may accumulate owing to airborne contamination from vehicle emissions and mobile exhausts [51]. The use of cadmium-rich fertilizers, the disposal of municipal garbage and irrigation with contaminated water are the main causes of heavy metal contamination in rice [52]. In this respect, Pescod [53] pointed out that the threshold values of heavy metals in irrigation water leading to crop damage are 2000 µg L^−1^ for Zn, 200 µg L^−1^ for Cu, 5000 µg L^−1^ for Fe, 200 µg L^−1^ for Mn, 200 µg L^−1^ for Ni, 5000 µg L^−1^ for Pb, and 10 µg L^−1^ for Cd. When compared to nearby soil samples irrigated using well water, soil samples from sewage-irrigated areas have higher concentrations of harmful metals such as arsenic (67%), lead (55%), nickel (84%), chromium (75%), cobalt (78%), and zinc (130%). This practice is ongoing in the Al-Ahsa Governorate [54,55]. The over-accumulation of heavy metals in soils, which eventually find their way into rice, can start a number of clinical issues in both humans and animals, including sperm motility [56], renal dysfunction [57], bone disorders [58], cirrhosis [59], encephalopathy [60], cardiovascular illnesses [61], and systemic malignancies [62]. Although the cadmium levels in rice are below the permitted limits, they are still extremely close to the MAC [52,55].

Similar results were observed in the present investigation, and the As contents dissipated the most from the rice, followed by Pb and Sb (Figure 2). Although the Cd contents decreased more in the high-rice:water system (T_4_ and T_5_), the decrease was non-significant as compared to As and Pb. This fact has been explained by some researchers, stating that excess cooking water and the drainage of that excess water from the rice result in a reduction in arsenic [63,64]. Very few studies have been conducted on the fate of heavy metals under rice cooking conditions [20,63,65]. Gray et al. [66] noticed that metals are more easily removed from parboiled rice than brown rice. Liu et al. [67] reported that cadmium decreased by only 3%, whereas the lead content was decreased by 20% during rice cooking.

The arsenic contents decreased by approximately 55%; however, the arsenic EDI was still higher than the PMTDI (25.2 µg/day). This means that cooking rice in a high-water system is desirable to remove toxic elements from the rice. This is also a common practice in some households, where rice is boiled in excess water, unnecessary water is removed, and the rice is cooked to the desired degree [63]. In a recent study published in 2019, Saudi citizens in Narjan city consumed a higher quantity of rice, and the average rice intake per adult according to a food frequency questionnaire (FFQ) survey was reported to be 243 g/day [32]. These findings show that the high-water cooking of rice and drainage of excess water from the rice during cooking returned better results in terms of reducing the health risks. However, arsenic in brown rice may pose potential health hazards to brown rice consumers. The health risk increases when the metal intake comes from multiple sources in the form of combined exposure to metals [11,42,68]. In our previous study on dates, we calculated the health risks to Saudi citizens from the daily consumption of dates [11,29].

For centuries, red Hassawi rice has only been cultivated in the Al-Ahsa region of the Kingdom of Saudi Arabia. The local residents are used to eating brown Hassawi rice in their traditional way, usually taking about two servings over a week. Furthermore, Hassawi rice is also relished by indigenous people for traditional occasions and gatherings in a variety of ways. However, this trend is currently decreasing, and people are shifting toward more economical and viable options of rice varieties available in the region. However, more studies on other pathways should be carried out in order to assess human health risks. Based on the results obtained in this study, it can be suggested that measures must be taken to ensure the safety of local food in terms of contaminant levels, and to reduce the risks from all possible exposure pathways. Keeping all these considerations in view, it can be concluded that the daily intake of brown rice is low, and there are no health risks involved in eating brown Hassawi rice; rather, it can provide vital beneficial nutrients for human health.

## 5. Conclusions

The current study revealed that the arsenic and lead contents in all samples exceeded the maximum allowable limits (MALs) defined by the FAO/WHO, while the Cd contents did not surpass the MAL. The EDI results demonstrated that, with the exception of arsenic, Hassawi rice is safe for eating in relation to the preliminary maximum tolerable daily intake levels. The health risk index for arsenic also exceeded the threshold value (1.00). Cooking the rice in excess water and draining the extra water from the rice during cooking exerted a significant effect on all metals, except for Cd. Cooking the rice in the low-rice:water ratio system was found to be less effective in comparison to cooking the rice in the high-rice:water ratio system. However, policy makers should formulate laws that should be properly maintained to reduce environmental pollution, and more attention should be paid to the studied areas, focusing on the HRI. In addition, good health and zero hunger are amongst the main sustainable development goals (SDGs). Furthermore, in vivo studies should be conducted to obtain more accurate findings on which the states of metals and metalloids are more toxic, and chelators are used for the dissipation of metals and metalloids from food items.

## Figures and Tables

**Table 1:** Occurrence of some heavy metals (mg/kg) in Brown Hassawi raw rice.

Heavy Metals	Farm 1	Farm 2	Al-Hofuf Market	* MAL
As	0.43 ± 0.02 ab	0.48 ± 0.03 a	0.40 ± 0.019 b	0.2 [31]
Cd	0.30 ± 0.03 b	0.37 ± 0.02 a	0.37 ± 0.031 a	0.4 [31]
Pb	1.36 ± 0.02 a	1.35 ± 0.02 a	0.98 ± 0.013 b	0.3 [31]
Cr	0.30 ± 0.01 b	0.39 ± 0.01 a	0.38 ± 0.015 a	1.0 [33]
Sb	0.09 ± 0.01 c	0.26 ± 0.02 a	0.20 ± 0.024 b	-
Ni	0.99 ± 0.02 c	1.39 ± 0.03 a	1.20 ± 0.049 b	-

* MAL means maximum allowable limit; similar letters in a row are non-significant among each other (*p* > 0.05); -: limit is not available or not defined.

**Table 2:** Mean square of the effect of cooking conditions on the heavy metals of rice.

Source	DF	As	Cd	Pb	Cr	Sb	Ni
Treatment	5	0.037 **	0.001 ^NS^	0.107 **	0.001 *	0.003 **	0.009 **
Error	12	0.000	0.000	0.000	0.000	0.000	0.000

** highly significant (*p* value < 0.01), * significant (*p* value < 0.05), NS. non-significant (*p* value > 0.05).

**Table 3:** Estimated daily intake of heavy metals through rice consumption in relation to PMTDI.

Treatments	As	Cd	Pb	Cr	Sb	Ni
T_0_	77.11	60.59	218.86	62.53	41.63	224.53
T_1_	76.95	60.26	218.05	62.37	41.31	224.37
T_2_	74.36	60.59	211.25	59.62	39.04	213.19
T_3_	73.06	59.62	209.30	59.94	38.39	212.06
T_4_	49.73	58.16	165.08	57.83	30.29	209.30
T_5_	34.18	52.81	147.74	57.02	29.32	201.76
* PMTDI	30 [34]	70 [35]	250 [36]	7000 [37]	360 [38]	-

Estimated daily intake (µg/person/day/162 g rice); * provisional maximum tolerable daily intake (µg/person/day); -: not available.

**Table 4:** Health risk index (HRI) of rice consumption due to heavy metals intake.

Treatments	As	Cd	Pb	Cr	Sb	Ni
T_0_	3.62	0.85	0.86	0.00	0.15	0.16
T_1_	3.61	0.85	0.85	0.00	0.15	0.16
T_2_	3.49	0.85	0.83	0.00	0.14	0.15
T_3_	3.43	0.84	0.82	0.00	0.14	0.15
T_4_	2.34	0.82	0.65	0.00	0.11	0.15
T_5_	1.61	0.74	0.58	0.00	0.10	0.14
* Oral Ref. Dose [31]	0.0003	0.001	0.004	1.50	0.004	0.02

* oral ref. dose (mg/kg/day).

## Data Availability

Not applicable.
